# Cardiovascular Surgeons’ Medical Perspectives Regarding Social Media Usage: a Survey Analysis

**DOI:** 10.21470/1678-9741-2020-0513

**Published:** 2022

**Authors:** Veysel Başar, Fatih Öztürk, Emre Kubat, Hakan Hançer, Ferit Çiçekçioğlu, Mehmed Yanartaş

**Affiliations:** 1 Department of Cardiovascular Surgery, Kartal Koşuyolu Yüksek İhtisas Training and Research Hospital, Istanbul, Turkey.; 2 Department of Cardiovascular Surgery, Marmara University, Pendik Training and Research Hospital, Istanbul, Turkey.; 3 Department of Cardiovascular Surgery, Gülhane Training and Research Hospital, Ankara, Turkey.; 4 Department of Cardiovascular Surgery, Bozok University, Faculty of Medicine, Yozgat, Turkey.

**Keywords:** Social Media, Public Health, Communication, Internet, Cardiac Surgery, Surgeons

## Abstract

**Introduction:**

We aimed to evaluate the use of social media among cardiovascular surgery specialists and their respective perspectives.

**Methods:**

In total, 173 cardiovascular surgeons were reached through an online survey. The surgeons surveyed were cardiovascular surgery specialists. The questionnaire consisted of 33 questions, including closed-ended and open-ended questions about social media.

**Results:**

We found that 73.4% of the participants think that social media facilitates the communication of the patient with the doctor, and 87.9% think that social media increases the publicity of the physician. Furthermore, 80.9% of the participants believe that informing through social media creates information pollution. We found that personal use of Instagram was more common in state hospital cardiac surgeons. The number of patients who contacted surgeons in private hospital for surgery via social media were found to be statistically significant, and it was found that this group benefitted more economically.

**Conclusion:**

Social media usage rates of cardiovascular surgeons were found to be high. On the other hand, it was observed that the rate of surgeons who share medical content is low. However, half the cardiovascular surgeons who participated in the study believe that their colleagues do not fully comply with the ethical rules in medical sharing.

**Table:** Abbreviations, acronyms & symbols

COVID-19	= Coronavirus disease 2019

## INTRODUCTION

The patient-doctor relationship has a history of 2,500 years. In the historical process, this relationship started with the “doctor-centered” approach, in which the doctor decided on behalf of the patient, and continued with the “patient autonomy” approach since the 1950s. Today, a synthesis of these two approaches is used. The change that began in the patient-doctor relationship in 1950s has gained a more revolutionary character in the social media age. Patients use social media, as well as the Internet, to research health and disease issues, treatment options, and information about doctors. Social media also changes the connection between doctors and patients and facilitates the interaction between the patient and the doctor by offering a two-way dialogue. It has also become a common platform for doctors to disseminate scientific and current developments to a wider audience^[1]^.

Currently, access to information is getting easier. Social media, which seems to increase social communication among people, helps to share information, facilitate communication between patients and physicians, and even increase earnings. However, it does not seem possible to say that all physicians are equally aware of this potential of the social media. In addition, using the social media to ease access to physicians can also increase the workload of surgeons who already spend a lot of time treating patients and can potentially restrict their private lives. A study conducted among plastic surgeons in the United Kingdom found that 36.2% of plastic surgeons used social media^[2]^. In the literature, no data is available on the use of social media by cardiovascular surgeons in Turkey.

In this study, we aimed to evaluate the use of social media by cardiovascular surgery specialists and their respective perspectives.

## METHODS

In total, 173 cardiovascular surgeons working in private and public hospitals were reached through an online survey. The surgeons surveyed were cardiovascular surgery specialists. All cardiovascular surgeons who participated in the study were divided into two groups in data analysis. The first group was formed by cardiovascular surgeons working in a private hospital and the second group by cardiovascular surgeons working in a state hospital. In order to understand whether there is a difference in terms of the answers given among surgeons with and without academic title, the surgeons who use social media were grouped again as academic surgeons and non-academic surgeons, and the results were reevaluated.

The questionnaire consisted of 33 questions, including closed-ended and open-ended questions (Supplement 1). Four of these questions evaluated age, gender, institution, and whether the surgeon is academic or not. Thirteen of the questions inquired the status of social media use, such as the status of active personal social media use, duration of the use, and whether they received professional support for their account or not. The remaining 16 questions evaluated the opinions of the participants on the use of social media. The question of getting professional support for social media account is defined as whether or not any support is received from any private company. The study protocol was approved by the local Ethics Committee (date: 12/12/2019, Nº. 2019.7/29-245). The study was conducted in accordance with the principles of the Declaration of Helsinki. A written informed consent was obtained from all participants.

### Statistical Analysis

Statistical analysis was performed using the SPSS Inc. Released 2008, SPSS Statistics for Windows, version 17.0, Chicago: SPSS Inc. software. In the descriptive statistics, categorical variables are shown as numbers and percentages, and numerical variables as means, standard deviations, medians, minimum and maximum values, and interquartile intervals. The suitability of the numerical variables to normal distribution was evaluated by the Kolmogorov-Smirnov test. The Student’s *t*-test was used to analyze significant differences between normally distributed data, while the Mann-Whitney U test was used to analyze non-normally data. The Chi-square and Fisher’s exact tests were used for the analysis of categorical variables. A type 1 error level < 5% (*P*<0.05) was considered statistically significant.

## RESULTS

**Fig. 1:**
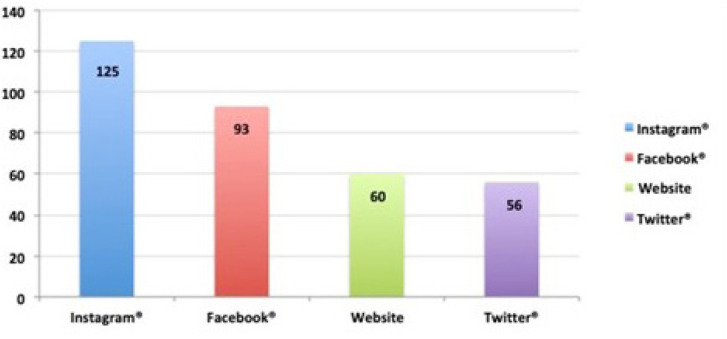
Number of surgeons using social media according to their platforms.

Of the 173 cardiovascular surgeons, 155 (89.6%) were male. When the titles were analyzed, the results showed that 116 (67.1%) of them were specialists, 34 (19.6%) were associate professors, and 23 (13.3%) were professors. In total, 127 (73.4%) of the participants were working in public hospitals and 46 (26.6%) were working in private hospitals. Of the 173 participants, 18 (10.4%) did not use any social media tools, while 80.6% (125 surgeons) of the 155 participants using social media indicated Instagram® as their most frequently used social media tool (Figure 1). There was no statistical difference between the status of social media usage in terms of age (43.6±7.7, 41.1±7.3 years, respectively; *P*=0.88). The use of a professional account, the frequency of sharing medical information, the rate of receiving professional support, and the number of pre-surgery referral to the social media were analyzed. While the rate of surgeons who promoted their services through social media was 14.8% (23 surgeons), the rate of surgeons who stated that they made scientific contributions was 47.7% (74 surgeons). Fifty-five (35.4%) of the participants communicated through the social media, while 15 (9.7%) stated that they were exposed to verbal violence at least once. While 127 (73.4%) of all the participants thought that social media facilitated patients’ access to physicians, 91 (52.6%) did not find it safe to use social media. Eighty-eight (50.9%) of the participants thought that surgeons’ social media usage increased patient satisfaction, and 152 (87.9%) thought that surgeons increased their publicity through the social media. Furthermore, 94 (54.3%) thought that they increased their job opportunities. One hundred and eleven (64.2%) of the participants thought that social media created unfair competition among surgeons. Eighty-nine (51.4%) of the participants did not comply with ethical rules and 29 (16.8%) of them thought it was against the principles of the protection of personal data and privacy. The majority of the participants thought that the use of social media increased institution-based publicity and that a significant portion of usage caused information pollution and should be controlled (Table 1).

**Table 1:** Results among the cardiovascular surgeons using social media.

	n=155
Social media usage frequency (average hour/day)	1.88±1.74
Account type/open account, n (%)	90 (58%)
Sharing frequency (pieces/week)	2.86±9.21
Using professional account, n (%)	33 (21.3%)
Medical sharing, n (%)	68 (43.9%)
Receiving professional support, n (%)	9 (5.8%)
Application for surgery through social media, n (%)	41 (26.5%)
Average application number from social media (units/month)	2.22±1.60
Social media use has economic contribution, n (%)	22 (14.2%)
Social media use has a scientific contribution, n (%)	73 (47.1%)
Communicating with patient through social media, n (%)	54 (34.8%)
Exposed to verbal violence in social media, n (%)	15 (9.7%)

Between private and public hospitals, Instagram® usage, occupational account usage, professional support, and economic contributions were found to be statistically significant in favor of private hospitals (*P*=0.009), while the other parameters were not statistically significant (Table 2).

**Table 2:** Results among cardiovascular surgeons according to the hospital.

	State hospital (n= 127)	Private hospital (n=46)	*P*-value
Age (years)	40.6±7.2	42.6±7.5	0.6^a^
Social media usage, n (%)	116 (91.3%)	39 (84.8%)	0.259^b^
Instagram®, n (%)	88 (75.9%)	37 (94.9%)	0.009^b^
Twitter®, n (%)	39 (33.6%)	17 (43.6%)	0.262^b^
Account type			0.002^b^
Open account, n (%)	58 (50.4%)	31 (79.5%)	
Hidden account, n (%)	57 (49.6%)	8 (20.5%)	
Frequency of sharing (median; min-max)	2.88±10.1	2.80±5.97	0.051^c^
(pieces/week)	(1; 0-100)	(1; 0-30)	
Professional account use, n (%)	17 (14.8%)	16 (41.0%)	0.001^b^
Medical sharing, n (%)	45 (39.1%)	23 (59.0%)	0.031^b^
Receiving professional support, n (%)	3 (2.6%)	6 (15.4%)	0.009^b^
Receiving the surgery application through social media, n (%)	25 (21.7%)	16 (41.0%)	0.019^b^
Surgery application through social media field (number/month) (median; min-max)	2.24±1.73	2.18±1.42	0.89^c^
	(2; 0-7)	(2; 1-5)	
The use of social media has economic contributions, n (%)	11 (9.6%)	11 (28.2%)	0.004^b^
Social media use increases surgeon’s recognition, n (%)	111(87.4%)	41 (89.1%)	0.923^b^
Social media use increases surgeon’s job opportunity, n (%)	69 (54.3%)	25 (54.3%)	0.998^b^
Social media creates unfair competition among surgeons, n (%)	82 (64.6%)	29 (63.0%)	0.98^b^
The institution in which the surgeon works uses social media, n (%)	40 (31.5%)	22 (47.8%)	0.122^b^
Institutions must use social media, n (%)	88 (69.3%)	37 (80.4%)	0.043^b^

a Student’s t-test;

b Chi-square test;

c Mann-Whitney U test

In terms of the titles (surgeon *vs*. academic surgeon), pre-surgery referral to the social media use and exposure to verbal violence were found to be statistically significant in the group with academic titles (*P*=0.017), but no statistical difference was found in the other parameters (Table 3).

**Table 3:** Results among cardiovascular surgeons according to the working title.

	Specialist (n=116)	Academician (n=57)	P-value
Age (years)	37.9±5.4	48.1±5.8	0.266^a^
Social media usage, n (%)	106 (91.4%)	49 (86%)	0.273^b^
Instagram®, n (%)	84 (79.2%)	41 (83.7%)	0.516^b^
Twitter®, n (%)	33 (31.1%)	23 (46.3%)	0.057^b^
Account type			0.011^b^
Open account, n (%)	54 (50.9%)	35 (72.9%)	
Hidden account, n (%)	52 (49.1%)	13 (27.1%)	
Frequency of sharing (pieces/week)	3.03±10.53	2.47±5.34	0.167^c^
(median; min-max)	(1;0-100)	(1;0-35)	
Professional account use, n (%)	20 (18.9%)	13 (27.1%)	0.25^b^
Surgery application through social media field, n (%)	24 (22.6%)	17 (35.4%)	0.097^b^
Average surgery count (median; min-max)	2.14±1,72	2.32±1.44	0.52^c^
(number/month)	(2; 0-7)	(2; 1-5)	
The use of social media has economic contributions, n (%)	12 (11.3%)	10 (20.8%)	0.118^b^
The use of social media has scientific contribution, n (%)	50 (47.2%)	23 (47.9%)	0.931^b^
Exposure to verbal violence from social media, n (%)	6 (5.7%)	9 (18.8%)	0.017^b^
Medical sharing in social media provides information pollution, n (%)	92 (79.3%)	48 (84.2%)	0.161^b^
Medical shares in social media should be inspected, n (%)	89 (76.7%)	49 (86.0%)	0.249^b^

a Student’s t-test;

b Chi-square test;

c Mann-Whitney U test

Seventy surgeons (40.5%) who participated in the study stated that they watched videos on video sharing platforms like YouTube® before the operation. The majority of the surgeons who watched a video online preoperatively were found to be non-academic surgeons (n=54, 77.1%). Compared to academician surgeons, this rate was found to be statistically significantly higher (*P*=0.02). The number of surgeons who think that video sharing sites contribute to post-graduate education is 93 (52%). In addition, 68% of the surgeons that answered affirmatively to this question (n=70) were non-academic surgeons, and this rate was found to be statistically higher than that of academic surgeons (*P*=0.013).

## DISCUSSION

Since the first e-mail was sent, communication technologies have been constantly and dramatically changing. Social media facilitates access to readily available information and communication between health professionals^[3]^. The growth of this network has enabled surgeons to exchange information between colleagues in specific areas of interest and to facilitate easy exchange of information between patients and surgeons^[4]^.

Today, there are many social media networks with different usage styles. It is known that there are 330 million active Twitter users today^[5]^. In our study, Instagram® was the platform with the highest rate of users (80.6%). This may be related to the fact that Instagram® provides more visual-based sharing opportunities. Today, social media is also used as a branding tool. This allows the buyer to access the product at any time^[6]^. If the surgeon is compared to a brand, the patient can access the brand’s product that he or she wants through social media at any time. In order for a brand to market its product, it must be able to communicate the characteristics of the product to the buyer well.

Before the social media era, brands used different ways to promote their products, such as television programs, print and visual media, and billboards. The social media has created a new way for brands to promote their products to consumers^[7]^. We believe that the practice of presenting surgeons to patients along with their titles and procedures has become more common with social media and that it has transformed surgeons into a brand. In our study, a significant majority of the participants thought that social media facilitated the patient’s access to the physician and also increased the publicity of the physician. However, there are no concrete sales of products to evaluate the surgeon as a brand. In addition, due to patient privacy and ethical and moral values, the advertising materials surgeons can offer to the recipients are limited.

Sullivan describes six basic features that must be found in a brand. A brand should be inspiring, entertaining, encouraging new ideas, confer status, useful, and accessible^[6]^. When these tenets are applied to surgeons, improving accessibility to the surgeon will play an important role in improving the quality of patient treatment. In addition, sharing posts that are entertaining and informative, and that show the surgeon’s welcoming nature will increase the patients’ motivation to be treated. In a study of 100 patients undergoing open heart surgery, a significant proportion of the patients had signs of preoperative and postoperative anxiety and depression^[8]^. Although preoperative information is given to the patient about the surgical intervention, psychological support may not be available to the extent desired. At this stage, motivating and informative exchanges through social media and comments made by similar patients about physicians may comfort the patient psychologically.

Especially with the coronavirus disease 2019 (COVID-19) pandemic, virtually in all medical branches, changes have started to occur in patient examination and treatment^[9]^. To this end, telehealth systems, where patients can communicate with physicians online, have gained popularity. These systems are especially an alternative for treatment and follow-up of patients with a chronic disease. It is suggested that such online communication tools increase compliance with the medical recommendations given to the patient^[10]^. Nevertheless, it should be noted that patients can record doctors while using this type of communication tool, and it is recommended to make careful comments while communicating with the patient in order to prevent legal problems that may occur in the future^[11]^. We did not focus on online systems, such as telehealth, as we solely focused on the use of the social media by cardiovascular surgeons.

In our country, Personal Data Protection Law draws the limits of the patient-doctor relationship. However, the standards for cardiovascular surgeons interacting with patients and with other healthcare professionals are provided by the American Association for Thoracic Surgery and Society of Thoracic Surgeons code of ethics, and these guidelines are fully applicable to the use of social media. Some of the standards related to online interactions mentioned in these guidelines are patient well-being, patient autonomy, honesty, fairness, confidentiality, and privacy^[12,13]^. These guidelines advocate preventing online public interactions between doctors and their patients during active care. It was reported that, although very few doctors respond, many patients send online “friend” requests to their doctors^[14]^. For this reason, it is stated that cardiovascular surgeons must maintain appropriate personal and professional limits while interacting with patients online due to the necessity of professionalism principle^[12,13]^. As new technologies and practices such as social networks have been adopted, it is very important to protect the confidentiality of patient information, to respect patients, to establish trust for doctors and the overall medical profession, and to establish appropriate limits^[2]^. An important problem with communication in the use of social media is the potential of information to be accessible by everyone. For this reason, the patients’ medical records should be kept in high-security databases^[10,15]^. Consent must be obtained from the patient in order to use their personal data online^[12,13]^. In addition, e-mail or other electronic communications should only be used by physicians in an established patient-physician relationship and with patient consent. It is stated that documents related to patient-care communication should be included in the patient’s medical record^[12,13]^. Cardiovascular surgeons usually record images/videos during the surgical operation for training and quality improvement purposes. Social media platforms make it easy to disseminate such recordings widely. However, it is stated that cardiovascular surgeons should be careful and make informed decisions when publishing them in professional accounts because the necessity of patient consent is emphasized in order to disseminate the medical records and images of the patients^[11-13]^. There may be posts that violate patient privacy, or it may cause unfair competition in terms of financial benefit. It is strongly emphasized that it is one of the physician’s responsibilities to protect the integrity of the line between the patient and the physician and to prevent ethical violations^[11-13]^. In the present study, 48.4% of the surgeons think that surgeons do not comply with ethical rules during the use of social media tools for professional purposes. Facilitating access to physicians through social media can lead to unwanted dialogue between the physician and the patient. Today, doctors are the most common target of violence on the social media in the field of health. In our study, 9.7% of the participants stated that they were exposed to verbal violence at least once through the social media. This situation is statistically more meaningful in the academic surgeons group.

Most of the surgeons that are in the post-graduate phase of surgical education (which is a lifelong process) prefer using video-sharing platforms to traditional textbooks to educate themselves. The laws regarding the privacy of personal data restrict this surgical education process regarding online education. In a previous study, 70% of the surgeons stated that they believed social media benefited professional development, while 22% stated that they preferred social media as the primary method of networking and communication with their colleagues^[16]^. Twitter® is the most popular form of social media currently used for health communication. Nevertheless, it is suggested that social media is not a suitable tool to share healthcare information, and that there is potential for false information, conflicting advice, and non-professionalization. However, communication opportunities provided by the social media are also used to improve clinical education^[17,18]^. The social media is also used to connect health professionals in third world countries with medically advanced specialists. For example, surgical procedures can be posted on the Internet and questions can be asked in real time via Twitter®^[16]^. Thus, healthcare professionals can create a professional network to share medical information in a way that was never before possible in terms of speed and easiness^[19]^. Furthermore, sharing guidelines through social media or the web may contribute to post-graduate medical training in order to facilitate access to information^[20]^. It was observed that cardiovascular surgeons in the present study had limited use of Twitter®. In addition, national and international associations have recently focused on online training after the COVID-19 pandemic. However, since the social media process was evaluated in our study, questions on this matter were not included.

Not sufficient data exist in the literature to determine whether social media helps physicians economically. As a result of the rapidly developing technology, the use of widespread social media in healthcare as well as in many other areas is increasing^[11-13]^. With the increasing use of social media commercially among physicians, positive and negative results as well as ethical and legal questions arise in many respects^[11-13]^. Our results showed that surgeons working in private hospitals earned more than surgeons working in public hospitals with the help of social media use. This situation can be related to the fact that physicians working in public hospitals have more standard and limited income sources and physicians in private hospitals have more employment opportunities. There is no clear social and legal limit on physicians’ use of social media. This may lead to unfair competition among physicians in case of a source of patient-based income. It should be noted that social media tools are all commercial initiatives. In fact, 64.2% of the surgeons who participated in our study thought that the use of social media caused unfair competition among physicians.

### Limitations

There are some limitations to this study. Firstly, although the number of participants in the study is low, it accounts for about 20% of cardiac surgeons actively working in the country. Therefore, we think that the results give an idea about the general direction in our country. Another limitation is that the reason for the low number of private hospital physicians in the study can be attributed to the fact that cardiovascular surgeons are mostly working in state hospitals due to the fact that the treatments related to cardiovascular surgery in our country are mostly given through state hospitals.

## CONCLUSION

In conclusion, in our study, social media usage rates of cardiovascular surgeons were found to be high. However, it was observed that although the rate of surgeons who share medical content is low, this type of use was mostly done by doctors working in private hospitals. Despite all this, it was observed that the rates of getting support from a professional company are low in the use of social media accounts. In addition, it was also observed that social media increased the publicity of cardiovascular surgeons and made it easier for patients to reach physicians. However, half of the cardiovascular surgeons who participated in our study believe that their colleagues do not fully comply with the ethical rules in medical sharing. We believe that the social media will become more important tool of communication and advertise among all professions. On the other hand, most of the participants believe that informing through social media creates information pollution. Therefore, we think that the present paper may contribute to the creation of national rules that shape patient-physician boundaries and ethical standards during the use of social media along with more detailed studies in the future.

## Supplement - Survey

1. Age:
2. Gender:
3. What is your title?
  Specialists
  Associate Professor
  Professor
4. What type of institution you work for?
  State Hospital
  Private Hospital
5. Do you use social media tools?
  Yes
  No
6. If your answer is ‘Yes’, please identify,
  -Facebook
  -Twitter
  -Instagram
  -Web site
7. If you don't use, do you plan to use?
  Yes
  No
  I have no idea
  If your answer to the 5^th^ question is ‘No', skip the questions between 7 and 18!
8. How often do you use? (On Average, Hour/Day)
9. What type of account do you use?
  Open Account
  Hidden Account
10. How often do you share? (Average number of shares/week)
11. Do you have a medical professional account?
  Yes
  No
12. Do you make medical shares via social media?
  Yes
  No
13. Do you get professional support for social media?
  Yes
  No
14. Do you have a patient applying to you through social media and having surgery?
  Yes
  No
  If your answer is ‘Yes’, how often? (number/month)
15. Do you think the use of social media has an economic contribution to you (with an increase in patient count)?
  Yes
  No
16. Does your social media use have any scientific contribution to you?
  Yes
  No
17. Do you communicate with patients through social media?
  Yes
  No
18. Have you been exposed to verbal violence by patients or relatives through social media?
  Yes
  No
19. Do you find the use of social media safe?
  Yes
  No
  I have no idea
20. Do you think social media makes it easier for the patient to reach the doctor?
  Yes
  No
  I have no idea
21. Do you think that communicating with patients through social media increases patient satisfaction?
  Yes
  No
  I have no idea
22. Do you think that the use of social media increases the recognition of the surgeons?
  Yes
  No
  I have no idea
23. Do you think that the use of social media increases the job opportunities of cardiac surgeons?
  Yes
  No
  I have no idea
24. Do you think advertisements made through social media create unfair competition among surgeons?
  Yes
  No
  I have no idea
25. Do you find the social media use of cardiac surgeons ethical?
  Yes
  No
  I have no idea
26. Do you think that the photos or videos (pre/intra/post-operative) that the physician takes including the patients, are in line with the privacy of personal data.?
  Yes
  No
  I have no idea
27. Do you think that the use of social media along with the intensity of work in cardiac surgeons is sustainable? (Does it cause extra waste of time?)
  Yes
  No
  I have no idea
28. Do you think it is necessary to use social media on an institution basis?
  Yes
  No
  I have no idea
29. Does the institution you work with effectively use social media?
  Yes
  No
  I have no idea
30. Do you think that the use of social media by the surgeons contributes to the recognition of the institution where he/she works?
  Yes
  No
  I have no idea
31. Do you think that medical posts through social media create information pollution?
  Yes
  No
  I have no idea
32. Do you think that the professional accounts and medical shares of cardiac surgeons should be inspected?
  Yes
  No
  I have no idea
33. Before the surgeries which you’ve planned to perform; do you often watch similar surgical operations via video-sharing platforms like Youtube etc.?
  Yes
  No
34. Do you think that sharing and watching surgical videos via online platforms contribute positive effects on heart surgery residency and post-graduate education?
  Yes
  No

## References

[1] Thiorux JP, Krasemann KW (2011). Ethics; Theory and Practice. 11the.

[2] Stevens RJG, Hamilton NM, O’Donoghue JM, Davies MP (2012). The use of the internet and social software by plastic
surgeons. Eur J Plast Surg.

[3] Moorhead SA, Hazlett DE, Harrison L, Carroll JK, Irwin A, Hoving C (2013). A new dimension of health care: systematic review of the uses,
benefits, and limitations of social media for health
communication. J Med Internet Res.

[4] Bosslet GT, Torke AM, Hickman SE, Terry CL, Helft PR (2011). The patient-doctor relationship and online social networks:
results of a national survey. J Gen Intern Med.

[5] Clement J (2020). Number of monthly active Twitter users worldwide from 1st quarter
2010 to 1st quarter 2019. https://www.statista.com/statistics/282087/number-of-monthly-active-twitter-users/.

[6] Sullivan L, Bennett S, Boches E (2012). Hey, Whipple, squeeze this: The classic guide to creating great
ads.

[7] Humphries LS, Curl B, Song DH (2016). #SocialMedia for the academic plastic surgeon-elevating the
brand. Plast Reconstr Surg Glob Open.

[8] Younes O, Amer R, Fawzy H, Shama G (2019). Psychiatric disturbances in patients undergoing open-heart
surgery. Middle East Curr Psychiatry.

[9] Mavioğlu HL, Ünal EU, Aşkın G, Küçüker ŞA, Özatik MA (2020). Perioperative planning for cardiovascular operations in the
COVID-19 pandemic. Turk Gogus Kalp Damar Cerrahisi Derg.

[10] Farnan JM, Snyder Sulmasy L, Worster BK, Chaudhry HJ, Rhyne JA, Arora VM (2013). Online medical professionalism: patient and public relationships:
policy statement from the American college of physicians and the federation
of state medical boards. Ann Intern Med.

[11] TK Jr Varghese, Entwistle JW 3rd, Mayer JE, Moffatt-Bruce SD, Sade RM (2019). Cardiothoracic Ethics Forum. Ethical standards for cardiothoracic
surgeons' participation in social media. Ann Thorac Surg.

[12] American Association for
Thoracic Surgery (c2018). Code of Ethics.

[13] Society of Thoracic
Surgeons (c 2021). Code of Ethics.

[14] Moubarak G, Guiot A, Benhamou Y, Benhamou A, Hariri S (2011). Facebook activity of residents and fellows and its impact on the
doctor-patient relationship. J Med Ethics.

[15] Thompson LA, Dawson K, Ferdig R, Black EW, Boyer J, Coutts J (2008). The intersection of online social networking with medical
professionalism. J Gen Intern Med.

[16] Wagner JP, Cochran AL, Jones C, Gusani NJ, Varghese TK Jr, Attai DJ (2018). Professional use of social media among surgeons: results of a
multi-institutional study. J Surg Educ.

[17] Tang Y, Hew KF (2017). Using twitter for education: beneficial or simply a waste of
time?. Computers & Education.

[18] Thompson MA, Majhail NS, Wood WA, Perales MA, Chaboissier M (2015). Social media and the practicing hematologist: twitter 101 for the
busy healthcare provider. Curr Hematol Malig Rep.

[19] Choo EK, Ranney ML, Chan TM, Trueger NS, Walsh AE, Tegtmeyer K (2015). Twitter as a tool for communication and knowledge exchange in
academic medicine: a guide for skeptics and novices. Med Teach.

[20] Durukan AB, Ertugay S (2017). Variations in international normalized ratio applications among
Turkish cardiovascular surgeons: daily practice versus
Guidelines. Turk Gogus Kalp Dama.

